# Mixed-planting: A useful tool to build climate-resilient forests

**DOI:** 10.1093/plphys/kiaf051

**Published:** 2025-02-07

**Authors:** Hannah M McMillan

**Affiliations:** Assistant Features Editor, Plant Physiology, American Society of Plant Biologists; Department of Biology, Duke University, Durham, NC 27708, USA

In addition to deforestation for lumber and development, forests worldwide face significant threats due to climate change. Warm, dry air can hold more water than cooler air. Therefore, as temperatures increase and drought becomes more frequent, the vapor pressure deficit increases. Increased vapor pressure deficit in turn drives increased transpiration, which leads to cavitation and embolism of xylem vessels in trees and results in greater tree mortality (Grossiord et al. 2020; Hammond et al. 2022; Christian et al. 2023). Understanding variation in drought susceptibility across tree species in a warming climate is critical for forest management and restoration efforts.

However, studying the impacts of drought, elevated temperature, and increased transpiration across tree species is difficult for several reasons. First, some tree species are larger than others and have a higher rate of water use, making it difficult to ensure that species within the experiment experience the same level of drought. Normalizing drought levels is nearly impossible in a natural forest system; however, planting in controlled soil conditions can allow researchers to carefully monitor and equalize soil water content across species. While effective, this approach is labor intensive and, as a result, limits the experimental sample size. Second, trees are long-lived, and water use varies across the tree's lifetime. Selecting tree species of the same age from similar soil conditions that have experienced similar drought conditions across their lifetime in a natural forest presents a significant challenge. Performing controlled experiments in seedlings may overcome age-related differences in water use and drought history, but it is possible that water use and susceptibility to drought in seedlings does not reflect the impact of drought on mature trees.

**Figure 1.**
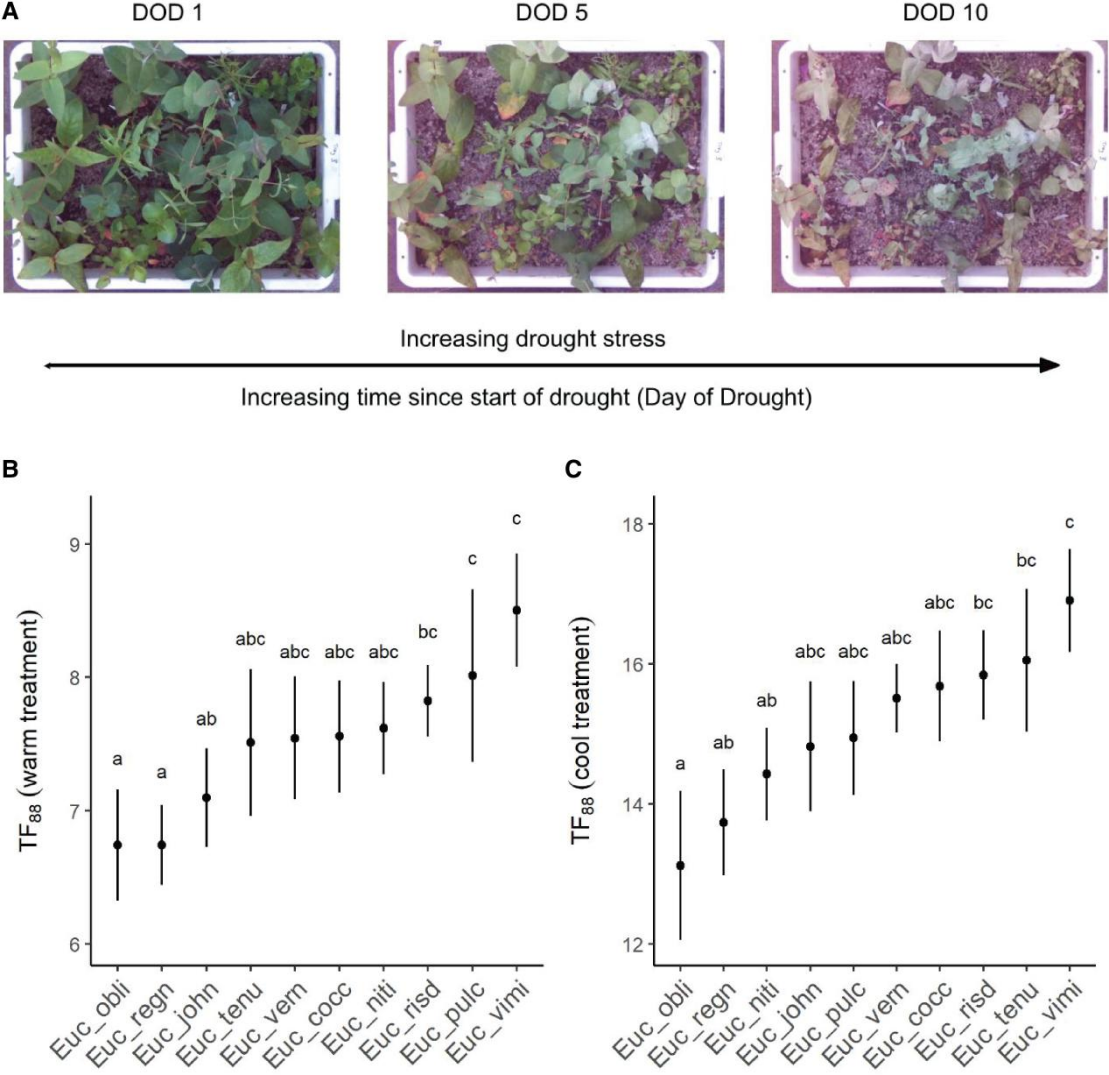
Mixed-planting reveals differences in seedling drought tolerance. **A)** Progression of drought stress in seedlings growing together in a single tray dried-down to death over 10 days under warm treatment conditions. DOD 1: all seedlings are hydrated; DOD 5: most seedlings show signs of leaf wilt; DOD 10: all seedlings have reached *Fv/Fm* values < 0.05 (representing a >88% decline from maximum). **B–C)** Differences among species in the time for seedling *Fv/Fm* to decline by 88% (*TF88*) under drought in warm conditions **(B)** or cool conditions **(C)**. Data points (solid circles) and whiskers represent species means and 95% confidence intervals, respectively. Differences in *TF88* between species in each experiment were tested using Anova. Significant differences (*P* ≤ 0.05) between species are denoted by different letters. Figure panels and legend modified from Blackman et al. (2024).

In this issue of *Plant Physiology*, Blackman et al. show that mixed-planting experiments using Eucalyptus seedlings can overcome challenges in controlling drought severity across species and reveal differences in drought tolerance across temperature conditions (Fig. 1A) (Blackman et al. 2024). Further, they provide critical evidence that species differences in seedling drought tolerance match differences observed in mature trees (Blackman et al. 2024). Experiments in seedlings can increase throughput and decrease the length of the experiment while still controlling for many variables. Results from Blackman et al. and future mixed-planting designs may be the key to understanding drought tolerance in trees quickly enough to protect forests from climate change.

Previous studies have shown that an 88% decline in chlorophyll fluorescence (*Fv/Fm*) is indicative of catastrophic xylem embolism and ultimately tree death (Tonet et al. 2023). Using time to an 88% decline in *Fv/Fm* (*TF88*) as a critical threshold to compare drought tolerance in their mixed-planting design, Blackman et al. compared 10 Eucalyptus species in warm and cool temperature conditions (Blackman et al. 2024). Under both warm and cool conditions, species showed significant differences in *TF88* (Fig. 1, B and C). *TF88* showed increased variation among species in the cool condition, providing better resolution to detect drought-tolerant versus drought-sensitive species (Fig. 1C). Drought recovery was not examined here; however, the authors note that *TF88* corresponded with the day recorded for meristem death, suggesting that *TF88* corresponds to lethal drought stress (Blackman et al. 2024).

Differences in seedling height impact water use and result in different rates of soil drying. These different rates mean that unless soil water content is carefully controlled, seedlings grown in separate pots will experience different drought severity. In the mixed-planting design, varied seedling height was observed across species (Blackman et al. 2024). However, because the seedlings shared soil, differences in height did not skew the dehydration rate across species. Indeed, pre-dawn water potential was consistent across seedlings during early drought conditions in the mixed-planting design (Blackman et al. 2024). Further, the mixed-planting design alleviates the technical burden of ensuring even dehydration across individual pots, allowing for larger-scale experimentation to test many different species and facilitate data collection sufficient to identify genomic and other -omic markers (Blackman et al. 2024; Candido-Ribeiro and Aitken 2024).

Perhaps most striking from the data presented in this study is that *TF88* measured in seedlings reflects the cavitation vulnerability measured in adult leaves on mature trees (Hartill et al. 2023; Blackman et al. 2024). This association indicates that differences in *TF88* observed in seedlings could reflect differences in drought tolerance in mature trees. Further, *TF88* is also linked to the mean annual precipitation at the species' sampling site, suggesting that the results in seedlings could reflect evolutionary adaptations of trees to specific climatic conditions (Blackman et al. 2024). Together, these conclusions suggest that results from seedlings can be extrapolated to drought tolerance in mature trees. As seedlings and mixed-planting designs provide a higher-throughput experimental design, this system opens the door to examine how different genetic loci are linked to drought tolerance. This type of information could help identify targets for engineering drought-tolerant trees or identify which species are ideally suited to our changing climate.

Overall, Blackman et al. show that mixed-planting experiments are a valuable addition to drought studies and control for many of the common pitfalls involved in experiments with individual pots (Blackman et al. 2024). They also reveal that results from seedlings correlate well with measurements of drought tolerance from mature trees, providing important evidence that data in seedlings from high-throughput assays can be used to extrapolate large-scale impacts on forests. These data and future mixed-planting experiments will be critical to inform species selection for forest restoration efforts in changing climatic conditions.
